# Dynamical comparison between Drosha and Dicer reveals functional motion similarities and dissimilarities

**DOI:** 10.1371/journal.pone.0226147

**Published:** 2019-12-10

**Authors:** Rotem Aharoni, Dror Tobi

**Affiliations:** 1 Department of Molecular Biology, Ariel University, Ariel, Israel; 2 Department of Computer Sciences, Ariel University, Ariel, Israel; Weizmann Institute of Science, ISRAEL

## Abstract

Drosha and Dicer are RNase III family members of classes II and III, respectively, which play a major role in the maturation of micro-RNAs. The two proteins share similar domain arrangement and overall fold despite no apparent sequence homology. The overall structural and catalytic reaction similarity of both proteins, on the one hand, and differences in the substrate and its binding mechanisms, on the other, suggest that both proteins also share dynamic similarities and dissimilarities. Since dynamics is essential for protein function, a comparison at their dynamics level is fundamental for a complete understanding of the overall relations between these proteins. In this study, we present a dynamical comparison between human Drosha and Giardia Dicer. Gaussian Network Model and Anisotropic Network Model modes of motion of the proteins are calculated. Dynamical comparison is performed using global and local dynamic programming algorithms for aligning modes of motion. These algorithms were recently developed based on the commonly used Needleman-Wunsch and Smith-Waterman algorithms for global and local sequence alignment. The slowest mode of Drosha is different from that of Dicer due to its more bended posture and allow the motion of the double-stranded RNA-binding domain toward and away from its substrate. Among the five slowest modes dynamics similarity exists only for the second slow mode of motion of Drosha and Dicer. In addition, high local dynamics similarity is observed at the catalytic domains, in the vicinity of the catalytic residues. The results suggest that the proteins exert a similar catalytic mechanism using similar motions, especially at the catalytic sites.

## Introduction

Sequence and structure alignment algorithms are long standing techniques to study proteins. Sequence alignment algorithms [1–3] identify residue conservation, while structure-based classification algorithms like CATH [4], SCOP [5], and DALI [6] provide a good overview of the entire protein structure universe. However, protein structures are dynamic rather than static, and understanding the relation between protein function and dynamics is fundamental for comprehending the protein structure–dynamics–function relationship. Such an understanding can stem from a comparison of the dynamics of related proteins or the same protein in different states. Traditionally, dynamical comparison of proteins was limited to conserved residues between related proteins [7–9]. In recent years, we [10–13] and others [14–17] have developed several tools and techniques for comparison of protein dynamics that are sequence and structure independent, contributing to the development of the field of comparative dynamics. The dynamical similarity between human Drosha and Giardia Dicer is presented in this study.

Dynamical comparison relies on analysis of low-frequency normal modes from coarse-grained elastic network models (ENM) such as the Gaussian Network Model (GNM) [18] or the Anisotropic Network Model (ANM) [19, 20]. This analysis was proven useful in unraveling the collective modes and, in particular, those at the low frequency end of the mode spectrum that underlie protein equilibrium dynamics [21]. With increasing availability of structural data for well-studied proteins in different forms (liganded, complexed, or free), there is increasing evidence in support of the correspondence between functional changes in structures observed in experiments and the global motions predicted by these coarse-grained analyses [22]. The low frequency modes (usually first 20) are concerned with functional motion and the similarity tend to decrease with higher energy modes. Comparison was made between principal component analysis modes obtained from micro- to milli- second full atomic molecular dynamics (MD) simulations [23, 24] and modes obtained from ANM. Close overlap was found between the principal modes of these two techniques, reinforcing normal mode analysis as a tool for exploring protein dynamics [25].

The Ribonuclease III (RNase III) family is a group of endoribonucleases that specifically degrades double-stranded RNA (dsRNA) at selected target sites, producing typical staggered ends containing 2-nt 3′-overhangs [26]. The cleavage is executed by a conserved catalytic site, which is placed in a unique endonuclease domain fold called a RNase III domain (RIIID) [27–29]. RNase III family members are divided into three classes based on domain organization. Drosha and Dicer are RNase III family members of classes II and III, respectively, which play a major role in the maturation of micro-RNAs (miRNAs) [30, 31]. In the nucleus, Drosha together with its cofactor DiGeorge Syndrome Critical Region 8 (DGCR8) constitute a complex known as Microprocessor. The complex excises a long primary transcript (pri-miRNA) to release hairpin shaped precursor-miRNA (pre-miRNA) of ~70 nucleotides in length. The pre-miRNA is subsequently cleaved at the cytoplasm by Dicer, yielding a miRNA duplex of ~22nt in length, when one strand of this duplex remains as a mature miRNA, while the other strand is degraded [26, 30–34].

**Fig 1:**
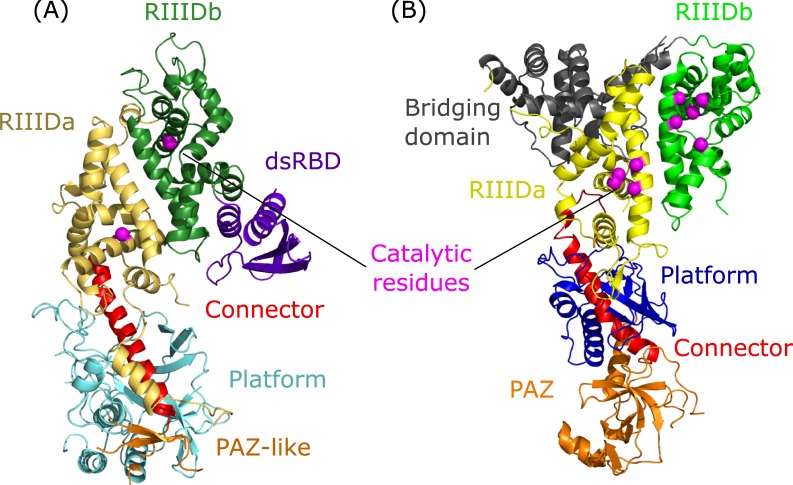
Structures of human Drosha and Giardia Dicer. Front view ribbon representation of the structure of (A) Drosha and (B) Dicer. The proteins are colored with similar colors for similar domains. Domains colors are as follow: N-terminal Platform domain (Drosha: cyan, Dicer: dark blue), putative PAZ-like and PAZ domains (Drosha and Dicer: orange), Connector (red), RIIIDa (Drosha: gold, Dicer: yellow), RIIIDb (Drosha: green-forest, Dicer: light green), Dicer Bridging domain (gray), Drosha dsRBD domain (purple). The catalytic residues of both RIIID domains are represented as magenta spheres.

Recent studies have found that human Drosha and Giardia Dicer (considered as the 'minimal' Dicer) share a similar domain arrangement and overall fold, besides the C-terminal component, despite no sequence homology [35, 36]. Both proteins have an elongated structure (Fig 1), with two upper RNase III Domains, RIIIDa and RIIIDb, that form a single processing center with two catalytic sites, one on each domain [33, 35, 37]. The RIIIDa is linked to a long α-helix called a Connector, which is encircled by the N-terminal residues that form the Platform domain. The Connector is followed by a bottom globular domain called PAZ for Dicer. The electron density of this region for Drosha was unclear due to structural flexibility, although the strong electron density blobs and predicted secondary structure imply a globular region that may adopt a PAZ-like fold (hence, referred to as the PAZ-like domain) [35]. The lower domains (Connector, Platform, and PAZ/PAZ-like) are collectively referred to as the central domain (CED). In addition to the similar domains, Drosha and Dicer have distinct domains. The two RIIIDs of Dicer are connected by a large helical domain called the Bridging domain, which is missing in Drosha. Another difference is that unlike Dicer, the RIIIDb of Drosha is bound through a broad and mostly hydrophobic interaction surface to a double-stranded RNA-binding domain (dsRBD). Drosha also has a specific conserved insertion of 67 residues that form two helical segments within its RIIIDa. The first helix (Bump helix) is half buried at a cleft formed by Platform and Connector. The other helix (mobile-basic helix or MB helix) is not shown in the structure, due to missing electron density [35, 36]. In addition, Drosha has at its N-terminal two domains: proline-rich (P-rich) and arginine/serine-rich (R/S-rich), which are dispensable for pri-miRNA processing activity in vitro [38]. Structurally, Drosha has a more bended posture compared with Dicer, which is primarily caused by the former’s extensive interaction surface between Connector and RIIIDa that is highly conserved and hydrophobic [35].

Drosha and Dicer act as "rulers" and "scissors" since they measure and cut at a fixed distance of each substrate [26, 36]. Drosha and Dicer catalytic domains (RIIIDs) are highly conserved among the RNase family and share a cleavage mechanism that involves two metal ions [29, 30, 33, 39]. In the single processing center formed by the intramolecularly dimerized RIIIDs, each catalytic site on each of the domains, RIIIDa and RIIIDb, cleaves one strand of an RNA duplex, the 3' and 5' strands, respectively. The Dicer action mechanism is better studied than Drosha's. The 2-nt, 3'-overhangs of a pre-miRNA are recognized and anchored to a specific pocket in the PAZ domain, while the 5' lies adjacent to the specific PAZ domain loop [30, 40]. The pre-miRNA twist approximately two helical turns towards the catalytic valley of the processing center that is located between the two RIIIDs. The distance between the PAZ and RIIIDs domains, in the case of Dicer, is 65Å and corresponds the length of 25bp, the length of the miRNA duplex. At the processing center, the pre-miRNA is cleaved, liberating a mature miRNA duplex [32, 41–43]. The ligand binding mechanism of Drosha is slightly different than that of Dicer. Drosha binds asymmetrically to the C-terminal tail (CTT) domain of two DGCR8 molecules, one on each RIIID. The two DGCR8 dimerize with the apical stem of pri-miRNA and so augment the substrate binding affinity of Drosha, and enhance Microprocessor fidelity [36, 38, 39, 44, 45]. Drosha interacts with the basal part. A recent study by Kwon et al. [35] presents an RNA-binding model of human Drosha with pri-miRNA. According to this model, the last base pair (bp) before the basal junction of pri-miRNA is adjacent to the Bump helix. The basal junction is where the dsRNA of the lower stem bifurcates to basal segments of single stranded RNA (ssRNA). The basal ssRNA segments of pri-miRNA are located near a positively charged conserved surface on the CED and extends to the PAZ-like domain, rather than the RIIIDs, due to a blockage against the dsRNA stem. The blockage is caused by bending of the Connector and the core of the Platform, toward the dsRNA, and due to the Bump helix with its surrounding loop. The pri-miRNA extends up from the basal junction (adjacent to the Bump helix) about one helical turn of ~28Å [38], which matches the length spanned by ~11bp, toward the catalytic site at RIIIDa. The dsRBD of Drosha interacts transiently with the dsRNA stem and helps to locate the processing centers ~11bp from the basal junction [39]. The pri-miRNA is then cleaved, yielding a 2-nt 3′-overhangs pre-miRNA, which is later processed by Dicer.

The overall structural and enzymatic reaction similarity of both proteins, on the one hand, and differences in the substrate and its binding mechanisms, on the other, suggests that both proteins share dynamics similarity and dissimilarities related to their common and unique functions. In this paper, we examine the dynamics similarity and dissimilarities of human Drosha and Giardia Dicer (hereafter referred to as Drosha and Dicer, respectively) by comparing their GNM and ANM modes of motions, using recently developed global [13] and local [46] mode alignment algorithms. We show that there is an overall low global dynamical similarity between the two proteins, while higher local dynamical similarity co-localizes with the catalytic domains.

## Methods

### Protein structures

Protein data bank (PDB) [47, 48] entries 5b16 [35] and 2ffl [40] of Drosha and Dicer, respectively, were used for dynamical comparison. The structures were superimposed using the Iterative magic fit tool of Swiss PDB viewer (SPDBV) [49] and their ANM modes of motion were calculated, as previously reported [20, 50, 51]. Each residue was represented by a single node positioned at its C^α^ atom and a cutoff distance of 15Å was used [52]. GNM modes were calculated as previously described [11, 18, 53] using C^α^ atom cutoff distance of 7.3Å. The first six ANM modes (and one for GNM modes) are so-called trivial modes with zero frequency (eigenvalue) and correspond to rigid-body rotation and translation. Therefore, first (slowest) mode refers to the first mode with non-zero eigenvalue.

The Drosha PDB entry includes two short helices of DGCR8 CTT, one on each RIIID, that are sufficient to interact with and stabilize the protein. In addition, some unknown (UNK) residues of the Platform and PAZ-like domains of Drosha are defined as hetero-atoms (HETATM). Both DGCR8 helices were found to have a negligible effect on the calculated modes of motion. Thus, the presented results were calculated without the two DGCR8 CTTs.

### GNM mode analysis

The motions along different GNM modes are found by eigenvalue decomposition of the connectivity matrix Γ = U Λ U^−1^ [18]. U is the orthogonal matrix of eigenvectors *u*_*k*_ of Γ and Λ is the diagonal matrix of the eigenvalues (*λ*_*k*_), 1 ≤ *k* ≤ N. The eigenvalues represent the frequencies of the *N*-1 non-zero GNM modes, and are organized in ascending order such that *λ*_1_≤*λ*_2_≤…≤*λ*_*N*−1_ and *λ*_*N*_ = 0. The *i*th element (*u*_*k*_)_*i*_ of the *k*th eigenvector describes the fluctuation (deformation) of residue *i* from its equilibrium position along the *k*th principal coordinate. The MSF of residue *i* can be rewritten as a weighted sum of the square fluctuations driven by all modes as:

$$\langle \Delta {\vec{R}}_{i}^{2}\rangle =\sum_{k}{\left[\Delta {\vec{R}}_{i}^{2}\right]}_{k}=3k_{B}T/\gamma \sum_{k}\left[\lambda_{k}^{-1}{\left({\vec{u}}_{k}\right)}_{i}^{2}\right]$$ (1)

GNM enables us to predict the relative *sizes* of motions accessed by different modes, not their *directions*, *the GNM fluctuations being isotropic by definition*. High fluctuations in absolute values correspond to protein structural regions with high mobility and vice versa. Regions with opposite GNM sign move in anti-correlated manners. Hinge is defined as the region where the fluctuations change sign. The directions of collective motions can be characterized by the Anisotropic Network Model (ANM) [20].

The fluctuations predicted by the GNM are isotropic and hence there is no information on the ‘directions’ of motion in the calculated modes, just their sizes and sign. In each mode, residues with opposite signs are moving in an anti-correlated manner. High (low) absolute fluctuation values indicate high (low) mobility. Hinge regions are defined as residues where the mode signs change. Regions where no more than three residues were of opposite signs were not considered as hinges.

### ANM mode alignment matrix

The ANM analysis describes motions of the proteins in Cartesian space and hence include information on the direction and the size of the motion. The commonly used Needleman–Wunsch [1] and Smith–Waterman [2] algorithms for global and local sequence alignment were modified to align ANM modes with few modifications. ANM mode analysis results in a set of vectors $\left\{\vec{U}\right\}$ describing the deformation of residues from their equilibrium position (native structure) in Cartesian space. Let $\vec{U_{i}^{k}}$ be the deformation vector of residue *i* in mode *k* of one protein and $\vec{V_{j}^{l}}$ the deformation vector of residue *j* in mode *l* of another protein. The score for residues *i* and *j* used for calculation of the alignment matrix of modes *k* and *l* is defined as:

$$S_{ij}=\frac{{\vec{U}}_{i}^{k}∙{\vec{V}}_{j}^{l}}{\left|{\vec{U}}_{i}^{k}||{\vec{V}}_{j}^{l}\right|}-C\;where\;0\leq C\leq 1$$ (2)

*S*_*ij*_ will be positive if their cosine value is greater than C that is the two deformation vectors pointing in the same direction and negative if their cosine is smaller than C. Here we used C = 0.7 radians (~40°) to define the threshold for vector similarity. In case of alignment of homologous or identical proteins, it is possible to guide the algorithm to prefer the matching of spatially close residues by applying distance constraints. Distance constraints were applied in the present work by modifying the alignment score *S*_*ij*_ as follows:

$$S_{ij}=\left\{ \begin{array}{l} \frac{{\vec{U}}_{i}^{k}∙{\vec{V}}_{j}^{l}}{\left|{\vec{U}}_{i}^{k}||{\vec{V}}_{j}^{l}\right|}-C,\;r_{ij}\leq R_{c}\\ -1\;,r_{ij}>R_{c} \end{array}\right.$$ (3)

where *r*_*ij*_ is the C^α^ distance between residues *i* and *j* and *R*_*c*_ is the cutoff distance set here to 20Å. Since the sign of the fluctuations in each mode is arbitrary, the alignment of two modes, *a* and *b*, is done twice: Once between the two original modes (*a* and *b*) and once between mode *a* and the negative of the second mode–*b*, with the best alignment (highest score) being used.

### ANM global mode alignment

The algorithm enables alignment of two modes even if their length is different as it creates insertions/deletions along the alignment in order to obtain the optimal alignment. Gap opening and extension penalties were set to 1.0 and 0.1. After the alignment process, in order to obtain a unified alignment score that is not dependent on the alignment length, an average alignment score (AAS) was calculated. The AAS is calculated as the average *S*_*ij*_ along the alignment with the exception of gap position and distance constraint regions that received a score of zero. The AAS range from zero (no match) to one (full match). Alignment was performed for the 40 slowest modes. This algorithm is an extension of our previous GNM modes alignment [10] and was reported previously in [13].

### ANM local mode alignment

For each pair from the *n* slowest aligned modes, an alignment matrix is created and the best non-overlapping (up to 200) gapless matches with minimal length of seven residues (gapless alignment of a single mode pair) are kept. The top *2n* matches (best scores) are selected and best *S*_*ij*_ is kept, for residues *i* and *j* of the first and second aligned proteins, for each mode combination [46]. The final residue dynamical similarity score (RDSS) of each residue is the sum of all its best (kept) *S*_*ij*_. The RDSS are then normalized to the range [0,99] to fit the B-factor scale of the protein structures. The PDB file B-factor values are replaced by the RDSS and best dynamically matching areas are visualized by the B-factor coloring option of the used viewer, Pymol [54]. The ability of the current algorithm to identify local dynamics similarity is demonstrated in our recent papers, where we present a detailed dynamical comparison between myoglobin and hemoglobin [46] and show its ability to perform dynamics-based clustering [55].

## Results

### Structural similarity of Drosha and Dicer

Human Drosha and Giardia Dicer are not apparent homologous proteins (global and local sequence similarity of 26.0% and 31.9%, respectively). However, recent studies [35, 36] found that both proteins share very similar domain arrangement and overall similar fold, apart from the C-terminal part (Fig 1).

**Fig 2:**
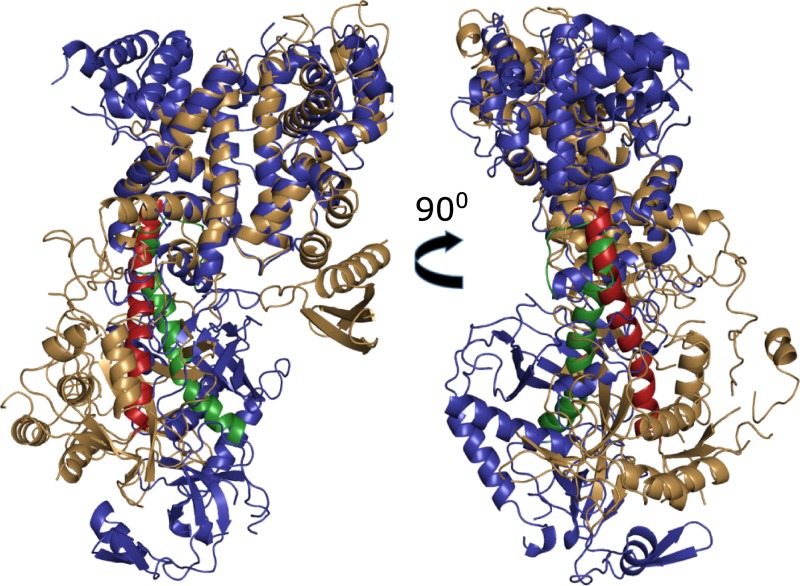
Superimposition of Drosha and Dicer. Front (left) and side (right) views of superimposition of Drosha (sand) upon Dicer (deep blue). Connector domain of Drosha is colored firebrick and of Dicer green-forest. Starting at the C-terminus of the Connector helix Drosha has more stooped posture compared with Dicer. The distance between the N-terminal of both Connector domains is 20.21Å, and the bending angle is ~30°.

Superimposing of Drosha and Dicer structures with SPDBV is depicted in Fig 2. Only 225 residues, mostly of the top part, out of 722 and 732 of Drosha and Dicer, respectively, were well superposed with RMSD = 1.31 Å. The top parts of the two proteins, containing the two RIIID domains are well superimposed (213 residues out of 421 and 444 of Drosha and Dicer, respectively), including residues at catalytic sites (see also S1 Fig. panel A, Supporting Information). Starting from the C-terminus of the Connector helix down to the PAZ/PAZ-like domain, Drosha has more stooped posture of the RIIID domains relative to the CED as compared with Dicer [35]. The distance between the N-terminal of both Connector domains (S271 of Drosha, I251 of Dicer) is 20.21Å, and the bending angle is ~30° as noted by Kwon et al. [35]. Nevertheless, the lower parts (CED) of the two proteins are also superimposable (S1 Fig. panel B), including the Connectors and the kink-inducing prolines (P864 and P266 for Drosha and Dicer, respectively) with high overlap degree (RMSD = 1.66 Å, 53 superposed residues out of 301 and 288 of Drosha and Dicer, respectively). Drosha's Connector is shorter than Dicer's (30 vs 38 residues), but its Platform is much bigger than Dicer's Platform (293 vs 124 residues). However, both Connector and Platform domains have similar orientation and show the same fold topology of both proteins. These general structural and folding similarities, despite no apparent homology suggest they share dynamical similarities.

### GNM slow modes of Drosha and Dicer

**Fig 3:**
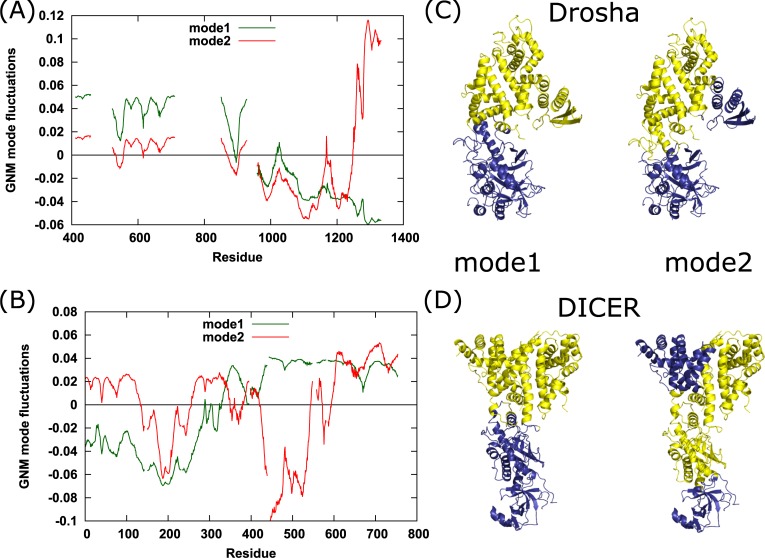
GNM first and second slow modes. Fluctuation values of the GNM first (green) and second (red) slowest modes of (A) Drosha and (B) Dicer. Residues with opposite signs are moving in anti-correlated manner and high (low) absolute fluctuation values indicate high (low) mobility. Residues at the crossover between positive and negative fluctuations act as hinges in the movement of the proteins. (C) Drosha and (D) Dicer structures colored according to the positive and negative signs of the first and second GNM slowest modes.

GNM analysis was used to calculate the slowest (global) motions of Drosha and Dicer. The two slowest modes of motions are presented in Fig 3 with the structures on the right colored according to the residue’s mode signs. The hinge sites of the first modes are at the crossover between residues C891-L892, T898-H899, L1018-D1019 and C1031-R1032 for Drosha and H328-T329 for Dicer. For both proteins, these hinge residues are located at the bottom of RIIIDa, the C-terminus of the Connector helix, and close to the interface between the Platform and RIIID domains. These findings suggest that the top and the bottom parts of each protein move in an anti-correlated manner. Drosha and Dicer structures are usually divided into two parts: a top part that includes the catalytic domains (RIIIDs) and a bottom part that includes the CED domains. The GNM slowest mode correlates with this division and indicates its dynamical role. The second GNM mode shows two hinge regions for both proteins. The first hinge of Drosha is located slightly below the axis of the hinge region shown by the first mode of GNM, and includes the lower half of the Connector, Platform, and PAZ-like domains (crossover between residues D532-G533, E555-E556, H869-I870 and N905-F906), whereas the second hinge separates the RIIIDs and dsRBD (P1247-R1248). As for Dicer, one hinge is located between the Platform and PAZ domains (L133-M134, L256-I257), and the other hinge resides between the Bridging domain and RIIIDa (A349-R350, L379-V380, I420-Y421, G543-F544, Q572-C573, and L593-A594). It should be noted that in both first GNM modes, residues D929 and T958 of Drosha have opposite signs. However, residues 930–957 are missing and belong to the MB-helix. Thus, the hinge region is located within this segment. Similarly, the PAZ-like domain is absent from the analysis.

### Drosha and Dicer mean square fluctuations profile

**Fig 4:**
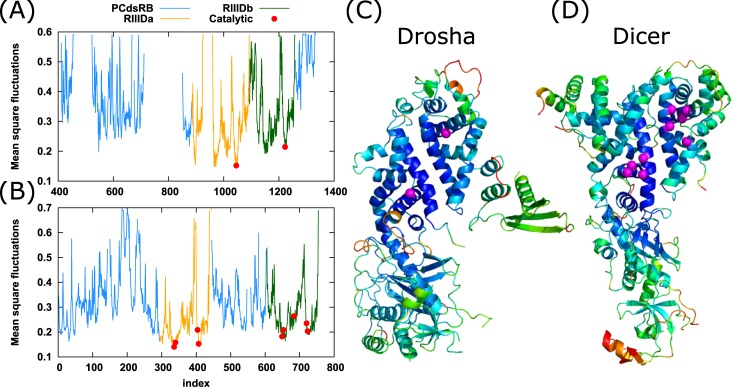
GNM mean-square fluctuations profile. Distribution of mean-square fluctuations (ordinate) as a function of residue index (abscissa) for (A) Drosha and (B) Dicer. Peaks represent the most mobile residues and minima the low mobility regions. Fragmented areas in the curve are UNK or missing residues in the PDB files. Curve colors are as follow: non catalytic domains (light blue; PCdsRB; platform, connector, dsRBD, bridging domain), RIIIDa (orange), RIIIDb (green). Catalytic residues are marked by filled red circles. (C) Drosha and (D) Dicer ribbon diagram is colored according to their MSF values. Warm and cold colors (red and blue scales, respectively) indicate most mobile and constrained regions, respectively. PCdsR designate: platform, connector, and dsRBD domains.

The above analysis enabled us to identify slow modes hinge regions. A complementary analysis of mean square fluctuations (MSF) indicates the mobility of each residue as calculated from all modes [18]. The MSF profile curves for Drosha and Dicer and the structures colored according to the residue mobility are depicted in Fig 4 and the catalytic residues are marked as red dots. The average MSF of RIIIDa is lower than that of RIIIDb (0.28 vs. 0.33 for Drosha, and 0.24 vs. 0.28 for Dicer), that is, RIIIDb is more mobile than RIIIDa. The catalytic residues tend to occupy the minima in the MSF curve, and thus they are relatively immobile. Dynamically, Drosha and Dicer exhibit very similar fluctuation profiles. Both catalytic domains have lower average mobility (0.39 for Drosha, and 0.33 for Dicer) relative to the non-catalytic domains (nonCD), while the lowest mobility is observed in the processing center cleft, including the two catalytic sites. Low mobility, though higher than that of catalytic domains, is also shown at the Connectors' C-terminus and Platforms' β-sheets of Dicer, compared with most of the Connector and Platform domains of Drosha. Higher mobility observed for PAZ than PAZ-like domains, probably resulting from the fact that most of the Paz-like residues are missing from the structure due to high thermal fluctuations.

### Drosha and Dicer global mode alignment

**Fig 5:**
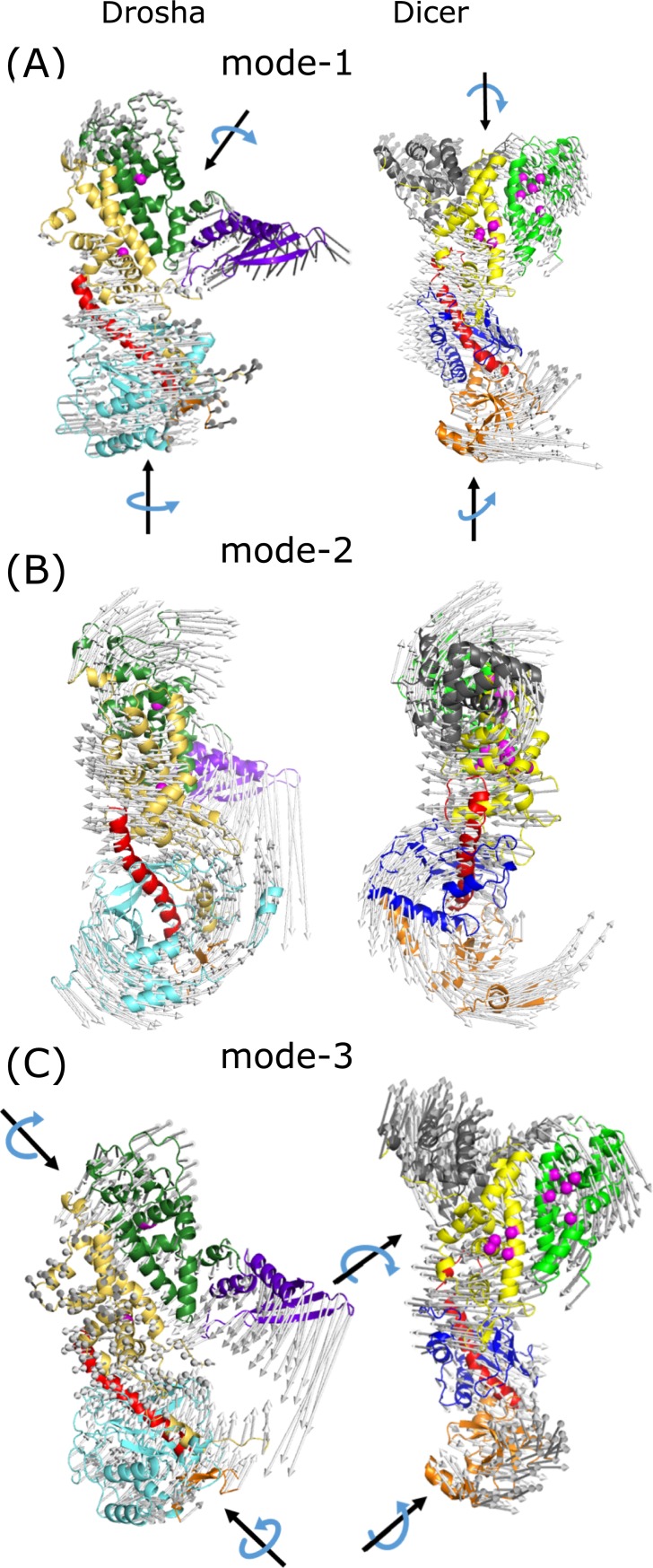
Porcupine plots of three slowest modes of Drosha and Dicer. Drosha (left) and Dicer (right) (A) first, (B) second, (C) and third ANM modes. The motion of the first and third modes is dissimilar for the two proteins, while the second mode shows reasonable similarity. Protein ribbon representation is colored according to the domain colors (see Fig 1 legend) and the arrows displaying the movement direction are colored light gray. Black arrows depict the rotation axis, blue arrows show the motion direction. Upper and lower parts of both proteins are moving in an anti-correlated circular shape. Porcupine plots were created using Modevectors [56].

The above calculation was performed using modes predicted by GNM, which are isotropic. More detailed dynamical information can be obtained by ANM modes of motion, which are anisotropic. ANM modes of the proteins were calculated and global mode alignment between the forty slowest modes of the two proteins were performed. The AAS matrix for alignments of the five slowest modes is presented in S2 Fig. Only the second mode of Drosha and Dicer show low to moderate similarity, with an AAS score of 0.33 while the rest of the modes are globally dissimilar. Even if global similarities are not observed, the protein's motion for at least the first five slowest modes is characterized by an anti-correlated movement of the upper domains and the CED, suggesting its functional importance. The motion is a semi-circular twisting and differs between the different modes as well as between both proteins in the rotation axis and direction (Fig 5). The change of the movement direction occurs at the hinge region between the RIIIDs and the CED, close to the lower catalytic site, in correlation with the location of the GNM identified hinge regions.

The first two or three slowest modes usually describe global functional motions that are to great biological interests [22]. Therefore, the dynamical difference of the slowest mode between the two proteins is of great importance. The slowest (first) modes of Drosha and Dicer show anti-correlated rotation of the top part against the CED (Fig 5A). However, the rotation axis of the Drosha’s top part is located between the RIIIDb and dsRBD, while the rotation center of Dicer’s top part is located above RIIIDa. The rotation axis of the CED is similar for Drosha and Dicer and located at the center of their domains. Movies capturing the slowest mode motion of Drosha and Dicer are provided in S1 and S2 Files, respectively. Drosha’s slowest mode results in a motion of the dsRBD toward the lower cleavage site at the binding cleft and a counter motion of the lower part of RIIIDa and CED domains. At the same time, RIIIDb and the top part of RIIIDa move backwards, away from dsRBD direction. The slowest mode of Dicer results in a sidewise anti-correlated twisting motion of the top part and the CED.

Drosha and Dicer’s motion according to their second mode is similar (Fig 5B). Despite existent differences between the motions, the global similarity of the two modes is pronounced. Additional viewpoints of this mode are shown in S2 Fig. panels B-E. The motion mainly describes the semi-circular, front-wise bending motion movement of the upper domains towards the lower domains (Platform and PAZ/PAZ-like). The hinge axis of both proteins is located between the RIIIDs and the CED, at the C-terminus of the Connector helix. The third mode of Drosha and Dicer is also dissimilar, due to differences in the rotation axis and motion direction of the top part and the CED domains (Fig 5C). Drosha's upper rotation axis is at the interface between the two RIIIDs, at the catalytic cleft, and the lower rotation axis is located at around PAZ-like domain and the Bump helix of RIIIDa. Whereas Dicer upper and lower rotation axes are at the interface between RIIIDa and the Bridging domain, and around the PAZ domain, respectively.

### Drosha and Dicer local mode alignment

**Fig 6:**
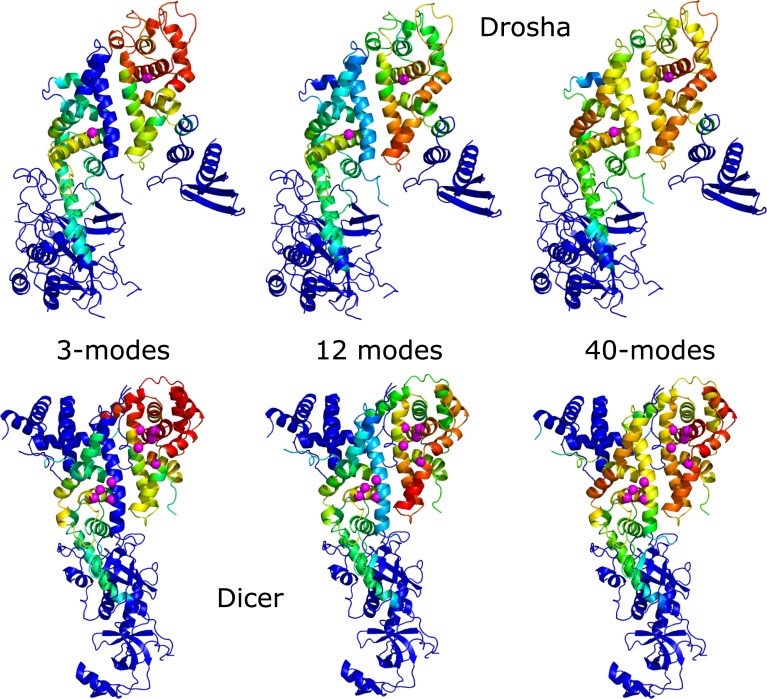
Local ANM modes alignment of Drosha and Dicer. Protein structures of Drosha (top) and Dicer (bottom) colored according to the RDSS calculated from local alignment of the three (left), twelve (middle), and forty (right) ANM slowest modes. Catalytic residues are represented as magenta spheres. Warm colors (high RDSS) represent high local dynamic similarity and cold colors (low RDSS) represent low dynamic similarity.

Global alignments revealed dynamical similarity between Drosha and Dicer of only one slow mode of each protein, although local similarities may still exist. Local mode alignments of the three, twelve, and forty slowest modes of the two proteins were performed. The higher the mode number, the higher the energetic cost of the protein to move along this mode, and the motions become more local and less global. Fig 6 shows the protein structures colored according to the RDSS. Warm colors (high RDSS) represent high local dynamic similarity (also referred here to as dynamical conservation) and cold colors (low RDSS) represent low dynamic similarity. In all three local mode alignments performed, moderate to high dynamic conservation (high RDSS) is observed at both RIIIDs and the Connector domain. RIIIDb is a little more mobile relative to RIIIDa (as predicted by MSF) and also tends to show higher conservation.

Each of the examined local mode alignments has a slightly different local dynamic similarity profile, although the three alignments show dynamical conservation at the same domains (two RIIIDs and Connector, as mentioned). In the first three modes, very high dynamic similarity is observed at the top part of the RIIIDb domain, while the RIIIDa helix located at the cleft between the two catalytic domains and, in the case of Drosha, the following helix, have very low dynamic similarity. At the twelve modes alignment, the dynamic similarity level of the top part of RIIIDb slightly declined, whereas the dynamic similarity of its lower part as well as the RIIIDa helix, that is in the cleft between the two RIIIDs, increased. Local mode alignment of forty modes shows that the highest RDSS residues concentrate at the areas containing the catalytic residues at each of the RIIIDs, parallel to rising of the dynamic similarity level of RIIIDa. Despite the few differences in the dynamic conservation profile of the three alignments, all three alignments show higher conservation in the vicinity of the catalytic residues for each catalytic domain at the processing center.

## Discussion

Drosha and Dicer are classes II and III members, respectively, of the RNase III protein family that play a key role in miRNA biogenesis [30, 31]. Functionally, both proteins act as a "ruler" and "scissors" as they measure and cut at a fixed distance of each substrate [26, 36]. The dsRNA substrate cleavage is carried out at the single processing center cleft containing two catalytic sites, generated by two intramolecularly dimerized strictly conserved catalytic domains (RIIIDa, RIIIDb). Previous studies report high similarity of domain arrangement and overall folding, despite few clear differences [35, 36]. The high structural resemblance of both proteins together with their similar function suggest they also share related dynamical similarities. However, since there are also differences between the proteins’ substrate and its binding mechanism, the dynamical comparison is expected to show dissimilarities related to unique functions. Therefore, comparison between Drosha and Dicer at their dynamics level is necessary to deepen the understanding of the relations between these proteins. To test this hypothesis, we analyzed the overall motion of each protein and dynamical comparison was performed using three methods: (i) GNM analysis, (ii) Global ANM modes alignment, and (iii) local ANM modes alignment. The last two methodologies were recently developed by us based on the commonly used sequence comparison algorithms, Needleman-Wunsch [1] and Smith-Waterman [2].

The GNM analysis shows that the least mobile region for both proteins is the substrate binding cleft (Fig 4). In addition, the catalytic residues are relatively immobile and tend to occupy the minima in the GNM MSF profile. These results are consistent with a previous study by Yang et al. [57] who showed that most of the catalytic residues in enzymes tend to have significantly smaller mobility compared to the average residues, probably to protect the delicate arrangement and orientation of these functional groups. A study by Macrae et al. [41] reported two flexible hinge regions at the Dicer structure. The first hinge is located between the Platform domain and RIIID domains. The induced positional shift is diffused along the Platform loop and the loop following the Connector helix, leading to bending of the RIIID domains towards the Platform, in addition to 5° rotation of the RIIID region around the long axis of the protein. The second hinge resides between the Platform and PAZ domains and is traced to strictly conserved Pro266. This flexible hinge induces a kink at the Connector helix, resulting in a shift of 5Å of the PAZ domain, and aids in directing the helix towards RIIIDa [40]. This kink-inducing proline is also found to be conserved in Drosha homologs (Pro864 in Drosha) [35]. The hinge found at the first GNM mode together with the lower hinge received at the second GNM mode for Dicer are consistent with those reported by Macrae et al. [40, 41]. The results also show a resemblance of the hinge locations of Drosha and Dicer, as well as the anti-correlated motion of the upper and lower parts.

GNM modes comparison can be viewed as a low-resolution dynamical comparison, since these modes provide information on the size of the fluctuations but not their directions. ANM modes provide both the direction and size of the motion, hence, can be viewed as a high-resolution dynamical comparison. Furthermore, each mode vectors $\left\{\vec{U}\right\}$ (see Methods) describing the deformation of residues from their equilibrium position of each mode are independent of each other. Therefore, both global and local alignment algorithms can be used to compare these modes. The results show partial dynamical similarity between Drosha and Dicer. Global mode alignments show a moderate similarity only of the second mode of Drosha and Dicer among the 40 slowest modes. Despite the existing similarities between Drosha and Dicer, there are significant differences between them, due to their distinct domains of each protein and the bent posture of Drosha. In addition, the substrates properties and their binding nature are likely to necessitate unique global dynamics for cleavage. The typical pri-miRNA cleaved by Drosha contains a dsRNA stem of ~11bp that is bound to the top part and flanked by single-stranded basal segments that are apparently bound to the CED domain [35]. However, the substrate of Dicer is a ~25bp dsRNA bound to the top part and the PAZ domain. Moreover, the substrate binding and cleavage mechanism of Drosha and Dicer are slightly different, with Drosha functioning as a part of the microprocessor complex [33, 39] while Dicer acts as an independent unit. Taking all the above together explains the dissimilar dynamics at the global level. Plausible explanation for difference in the global (first mode) dynamics of the two proteins is that since Dicer’s substrate is more rigid, bending motion of the first and second modes will result in separation of the cleaved dsRNA. However, since the ssRNA bound to Drosha’s CED domain are more flexible the slowest mode of Drosha evolve a stretching away movement of the CED domain with inward movement of the dsRBD as a more efficient motion that can separate the cleaved RNA products.

Although no significant global similarity was found, both Drosha and Dicer exhibit similar global motion trend, of bending the upper and lower domains toward each other, while moving in a semi-circular manner. Since this kind of movement is observed in the different modes of the two proteins it is probably of a functional significance of assisting the cleavage of each substrate. Rotating the substrate can impair its stability by generating high torsional stress or by stretching chemical bonds. Bending of the upper and lower domains towards each other together with the substrate can physically assist the cleavage mechanism.

As members of RNase III family, Drosha and Dicer catalytic domains are highly conserved and function with the same mechanism of dsRNA cleavage, although they have different substrates (pri- and pre- miRNA). Therefore, it is expected that the two proteins will have similar but not identical local dynamics. Local mode alignments show higher dynamic conservation at both RIIIDs, in the vicinity of the catalytic residues and at the Connector domain. The high dynamical conservation of the processing center indicates that Drosha and Dicer use similar local dynamics for the catalytic mechanism.

In this study, we present relatively new methodologies for dynamical comparison of proteins and exemplified the use of comparative dynamics as a new layer to study proteins by comparison. Sequence, structure, and dynamical comparison algorithms are three complementary layers to study proteins, and together give a more complete and accurate view of the relation between related proteins.

## Supporting information

S1 Fig Superimposition of Drosha and Dicer. (PDF)

S2 Fig ANM global mode alignment. (PDF)

S1 File Drosha’s slowest mode of motion movie. (MP4)

S2 File Dicer’s slowest mode of motion movie. (MP4)
