# Supplementary material for: Dynamical comparison between Drosha and Dicer reveals functional motion similarities and dissimilarities
Source: PLoS One. 2019 Dec 10;14(12):e0226147. doi: 10.1371/journal.pone.0226147 (PMC6903759; doi:10.1371/journal.pone.0226147)
Supplement: S1 Fig — (PDF) [file pone.0226147.s001.pdf]

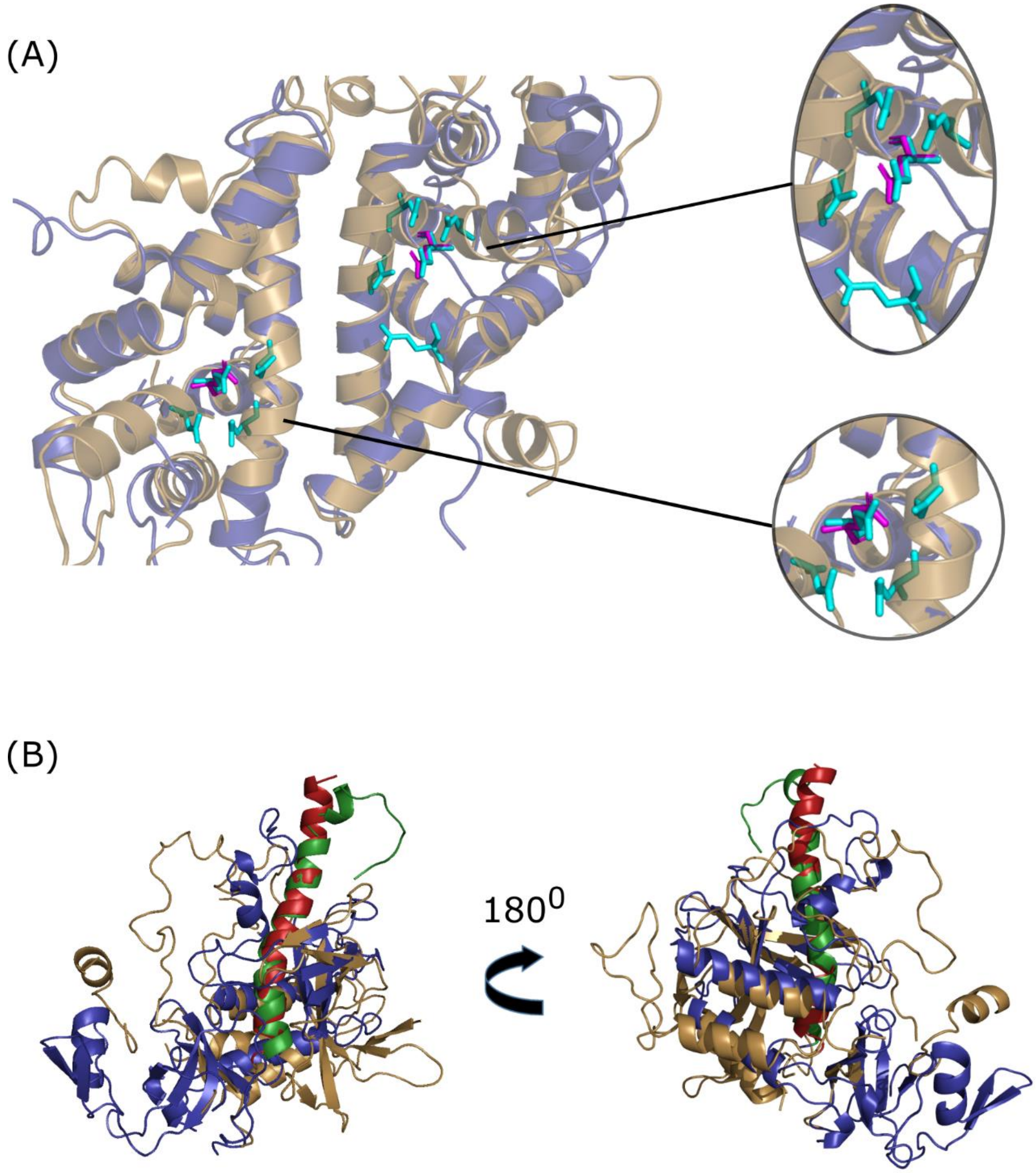

S1 Fig. Superimposition of Drosha and Dicer. (A) Superimposition of catalytic domains (RIIIDa, RIIIDb) of Drosha and Dicer, together with the catalytic residues. Upper and lower insets at the right are of RIIIDb and RIIIDa catalytic sites, respectively. (B) Back and front views of superimposition of CED domains (lower parts) of Drosha upon Dicer. Colors as the following: Drosha in sand, Dicer in deep blue, Catalytic residues in magenta (Drosha) or cyan (Dicer) sticks and Connector domains in firebrick (Drosha) or forest (Dicer).
