# Supplementary material for: Dynamical comparison between Drosha and Dicer reveals functional motion similarities and dissimilarities
Source: PLoS One. 2019 Dec 10;14(12):e0226147. doi: 10.1371/journal.pone.0226147 (PMC6903759; doi:10.1371/journal.pone.0226147)
Supplement: S2 Fig — (PDF) [file pone.0226147.s002.pdf]

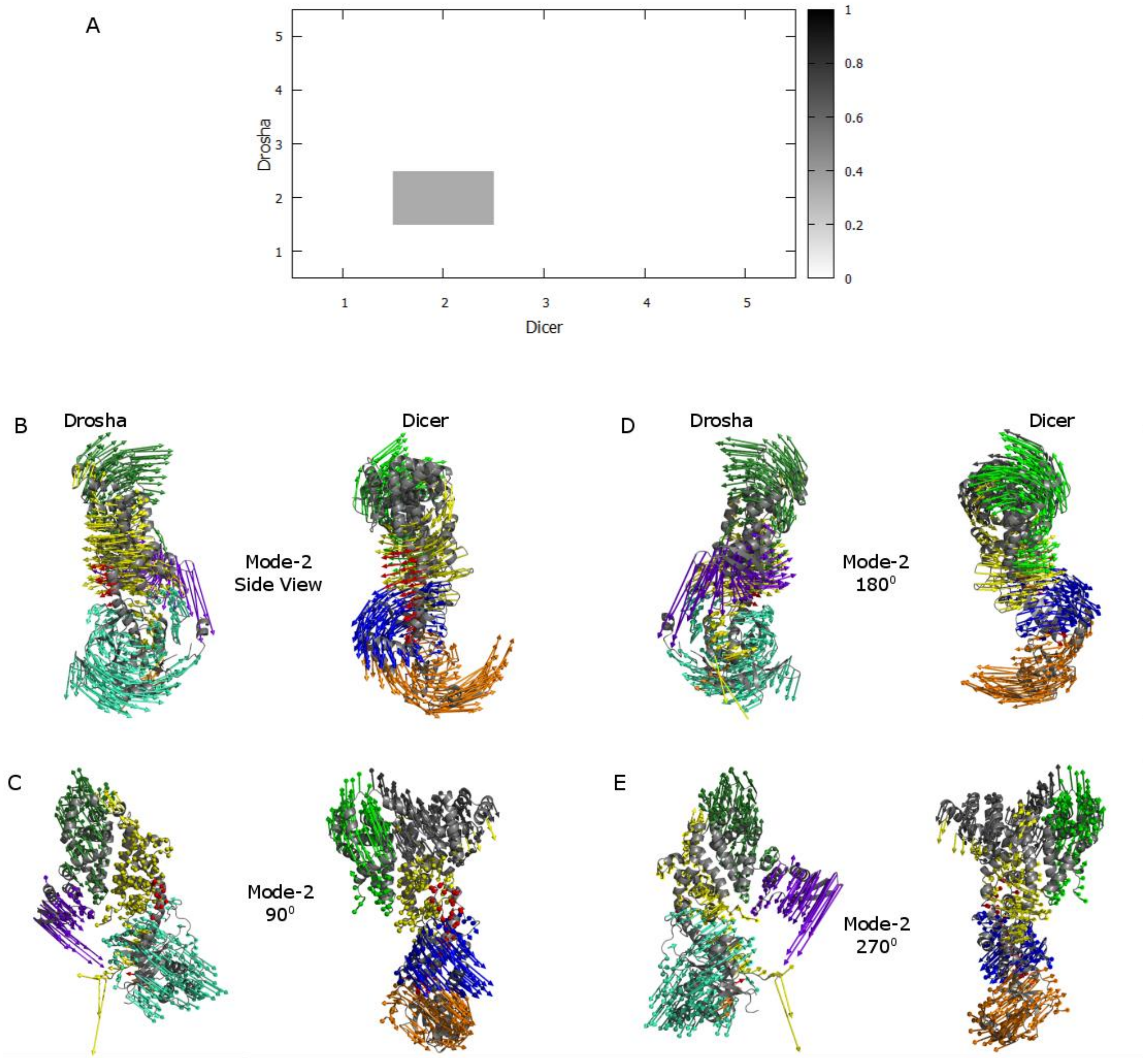

S2 Fig: ANM global mode alignment. (A) Alignment of the five ANM slowest modes of Drosha and Dicer. Out of global mode alignment calculations for the forty slowest modes, only the second mode of Drosha and Dicer show global dynamic similarity, with AAS score of 0.33. No similarity was observed for the rest of the modes (6 – 40) (B-E) Four different viewpoints of the movement of Drosha and Dicer second mode of motion depicted by porcupine plots. Viewing angles of the proteins are in respect to panel A displayed plot. Arrows pointing at the movement direction and are colored according to the domain in from which they point (detailed at Fig.1 legend).
